# Evidence-based interventions in dementia: A pragmatic cluster-randomised trial of an educational intervention to promote earlier recognition and response to dementia in primary care (EVIDEM-ED)

**DOI:** 10.1186/1745-6215-11-13

**Published:** 2010-02-10

**Authors:** Steve Iliffe, Jane Wilcock, Mark Griffin, Priya Jain, Ingela Thuné-Boyle, Tamar Koch, Frances Lefford

**Affiliations:** 1 Research Department of Primary Care and Population Health, University College London, Royal Free Campus, Rowland Hill St., London NW3 2PF, UK

## Abstract

**Background:**

The National Dementia Strategy seeks to enhance general practitioners' diagnostic and management skills in dementia. Early diagnosis in dementia within primary care is important as this allows those with dementia and their family care networks to engage with support services and plan for the future. There is, however, evidence that dementia remains under-detected and sub-optimally managed in general practice. An earlier unblinded, cluster randomised controlled study tested the effectiveness of educational interventions in improving detection rates and management of dementia in primary care. In this original trial, a computer decision support system and practice-based educational workshops were effective in improving rates of detecting dementia although not in changing clinical management. The challenge therefore is to find methods of changing clinical management. Our aim in this new trial is to test a customised educational intervention developed for general practice, promoting both earlier diagnosis and concordance with management guidelines.

**Design/Method:**

The customised educational intervention combines practice-based workshops and electronic support material. Its effectiveness will be tested in an unblinded cluster randomised controlled trial with a pre-post intervention design, with two arms; normal care versus the educational intervention. Twenty primary care practices have been recruited with the aim of gaining 200 patient participants. We will examine whether the intervention is effective, pragmatic and feasible within the primary care setting. Our primary outcome measure is an increase in the proportion of patients with dementia who receive at least two dementia-specific management reviews per year. We will also examine important secondary outcomes such as practice concordance with management guidelines and benefits to patients and carers in terms of quality of life and carer strain.

**Discussion:**

The EVIDEM-ED trial builds on the earlier study but the intervention is different in that it is specifically customised to the educational needs of each practice. If this trial is successful it could have implications for the implementation of the National Dementia Strategy.

**Trial registration:**

NCT00866099

## Background

Dementia presents many challenges for primary care. Early diagnosis is important as this allows those with dementia and their family care networks to engage with support services and plan for the future. These actions can relieve the significant psychological distress that people with dementia and close supporters may experience [1] and can also provide knowledge about the availability of medical and psycho-social support that can improved functioning and morale.

The main efforts to improve the identification and diagnosis of dementia should logically be targeted at primary care as this is the first point of contact in the health service for most individuals and their carers. There is, however, evidence that dementia remains under-detected and sub-optimally managed in general practice [2]. An educational intervention that could enhance clinical practice, improving the skills of practitioners in the recognition of and response to dementia syndrome could therefore be beneficial to people with dementia and their families; in addition it has the potential to improve the effectiveness of other health and social services by more timely and appropriate referral. The National Dementia Strategy [3] seeks to promote the professional development of general practitioners to enhance their diagnostic and management skills in dementia, making this trial particularly timely.

### Recognition and response

This project is based on an earlier portfolio of work which demonstrated that educational interventions can improve the recognition of dementia syndromes in general practice. The Alzheimer's Society Dykes Award RCT was an unblinded cluster randomised controlled study which tested the effectiveness of educational interventions in improving detection rates and management of dementia in primary care. A computer decision support system and practice-based educational workshops were effective in improving rates of detecting dementia although not in changing clinical management [4]. One challenge for the EVIDEM-ED trial is to develop an intervention that is grounded enough to fit into routine clinical practice and powerful enough to change the clinical management of patients with dementia in primary care.

### Changing practice

There are different barriers to changing clinical practice in different settings and at different times. Change may be more likely to occur if strategies are chosen to overcome identified barriers. Barriers can be related to the individual (e.g. uncertainty about the risks of a procedure); to social issues (e.g. peer pressure to perform a certain way) and to the organisation of services (e.g. no access to resources) [5]. Incentives to change also need to be identified and built into educational strategies [6]. Innovation in practice depends on the attributes of the new way of working that is being offered [7,8].

The development of an educational intervention needs to take into account the factors which may influence its effectiveness. These include personal factors such as learning styles; external factors such as caseload and demography of practices; confounding factors such as other clinical, educational or managerial demands; the skills of tutors or facilitators; and opportunities for learning from others [9]. Synthesising these lessons, the 'ideal' educational package would allow the practitioner to build upon existing clinical expertise and knowledge within a busy and demanding work schedule [10] and be flexible enough to accommodate old and new approaches to education [11]. Above all, such an educational package needs to be relevant to learners and enhance their problem-solving capacity by offering knowledge that can be applied in the normal milieu of practice as a form of 'soft technology', i.e. in the taken-for-granted skills that are brought to bear on routine clinical tasks [12]. In an evidence-based world, the construction of the 'ideal' educational intervention would require educationalists to work out *what *needs to be learned, *how *that learning can be facilitated and *in what forms *knowledge should be organised for maximum impact on clinical practice.

The necessary and sufficient basis for the development of an effective educational intervention appears to be the application of *propositional knowledge *of the kind generated by critical appraisal of the research literature with *process knowledge *acquired during work with patients. Two factors are likely to determine the structure of an educational package in which propositional knowledge is combined with case discussions. The first is the distribution of practitioners along a continuum from novice through advanced beginner, proficient, competent to expert [13]. The learning process should allow 'novices' to move towards proficiency and so should contain a hierarchy of complexity in the cases used to illustrate the application of propositional knowledge. However, since general practices contain doctors and nurses with different levels of experience and different agendas when approaching patient care, it must also allow the *competent *and *proficient *to expand their repertoire of skills without working on material that is already within their abilities. The advantage of learning in a mixed ability and mixed professional group is that peers and colleagues can be the most effective educators [14] and there is a particular benefit from learning from other disciplines [15].

In the first phase of this study, we used these insights to develop a flexible learning needs assessment tool that allows an educational intervention to be tailored to practice needs and the skills of the existing practice team. Further details of how this tailored intervention was developed are available elsewhere [16,17]. This intervention will be tested in the EVIDEM-ED randomised controlled trial.

### Aims and Objectives

Our aim is to develop and test an educational intervention for general practice, combining timely diagnosis and psychosocial support around the period of diagnosis with concordance with management guidelines [2,3].

The objectives of the study are:

1. To develop an educational intervention suitable for workplace use that has the potential to change management practice in dementia care amongst general practitioners and practice nurses.

2. As part of management in general practice, to include shared care guidelines for medication use by patients with dementia.

3. Developing and testing electronic resources that promote the above objectives.

## Design

### Trial design

**Figure 1:**
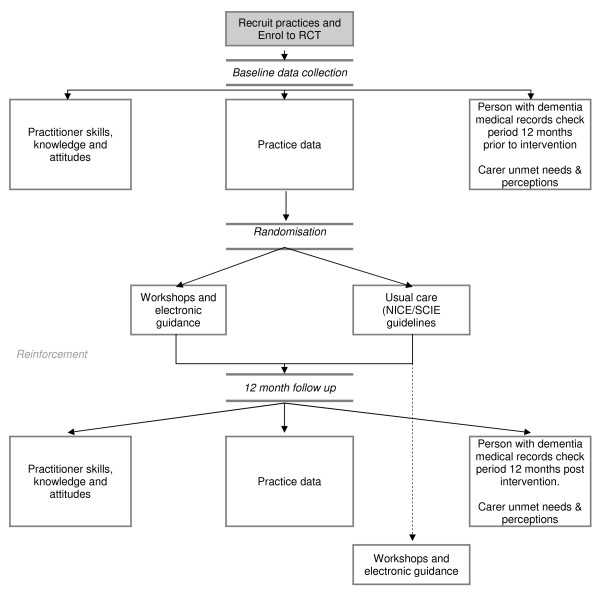
**The Consort EVIDEM-ED-Flowchart**.

The effectiveness of an educational intervention combining practice-based workshops and computer based support will be tested in a pragmatic unblinded cluster randomised controlled trial with a pre-post design and with two arms; normal care versus educational intervention (see consort diagram in Figure 1). The researchers will be aware of group allocation but carers and people with dementia will not be informed.

### Study setting

The study will take place within Primary care practices in the geographical care covered by the North Thames Dementias and Neurodegenerative Diseases Research Network (NT DeNDRoN); Metropolitan North London, Essex, Hertfordshire and Bedfordshire. Approval for the trial has been received from Southampton & South west Hampshire Research ethics Committee (A): reference 09/H0502/77

### Educational intervention

The educational intervention consists of practice based workshops with a tailored curriculum designed by a multidisciplinary expert group and supplemented by electronic resources. The educational interventions reflect different approachesto adult learning, namely workshops directly relevant to clinical practice, allowing learning to occur through peer reflection about real cases, and electronic resource materials suitable for 'real time' use in consultations.

#### Workshops

**Figure 2:**
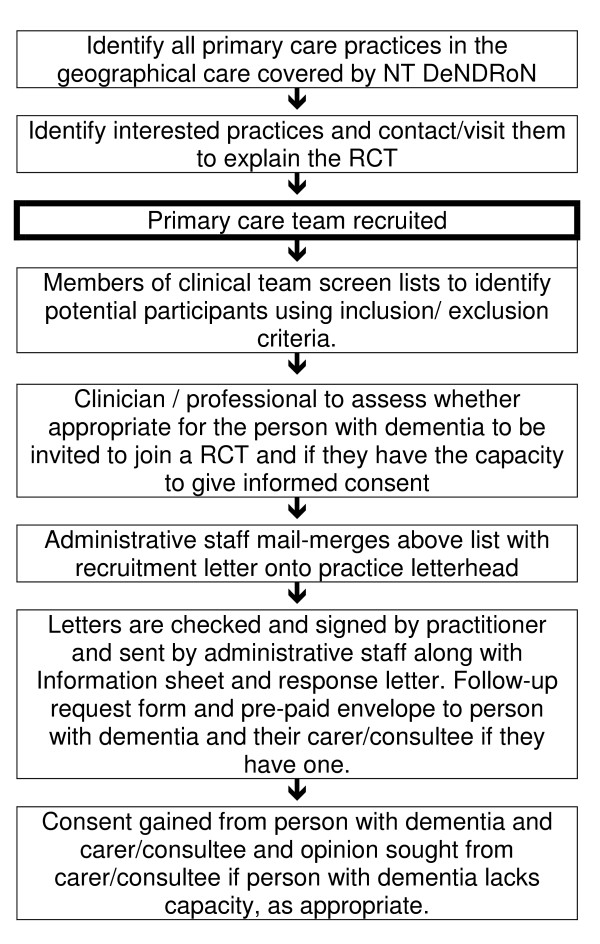
**The identification of participants and how these will be approached, recruited and consented/consulted about EVIDEM: ED**.

The EVIDEM programme has developed a workplace-based approach to training GPs in dementia diagnosis and management. The essential components of this approach are an 'educational needs assessment' and an 'educational prescription'. The educational needs assessment requires a one hour group meeting in the workplace, with the membership of the group determined by the work unit - in our case, the general practice. The facilitator of the group uses a standard checklist to elicit the strengths and weaknesses of the organisation's current practice with people with dementia (see Figure 2). Because inexperienced professionals often misjudge their learning needs, silences and absences in this discussion are interpreted by the facilitator. For example, it is possible for a general practice to discuss dementia care for most of the hour without mentioning carers' needs; the facilitator must point this out. The group can then identify the highest priority topics for learning and decide how it wishes those topics to be presented - as workshops, in an electronic format for self-directed learning, or with a printed manual (Appendix 1).

## Recruitment

### Primary Care practices

Interested general practices in the North Thames DeNDRoN area will be identified in collaboration with the local Primary Care Research Networks: the Primary Care Research Network-Greater London (PCRN-GL) and the Primary Care Research Network-East of England (PCRN-EoE). Practices will be contacted by the Trial research team, by letter and awareness-raising through general practice educational meetings and by regular newsletters.

#### Inclusion criteria

The inclusion criteria for practices in this study will be: 1) routine data collection from clinical encounters on electronic medical records and; 2) team commitment to participate in educational workshops held in the practice. (All staff working in the practice will be eligible to participate in the study.)

#### Exclusion criteria

Practices that do not routinely capture clinical data in electronic records will be excluded.

### Patients with dementia and their carers

#### Inclusion criteria

The inclusion criteria for people with dementia in this study will be a diagnosis or suspected diagnosis of dementia of any type, with no lower age limit.

#### Exclusion criteria

Exclusion criteria will be: 1) people who cannot speak English and for whom an interpreter cannot be located; 2) if the patient or carer are involved in concurrent research; 3) if the key professional feels that an approach to the person with dementia or their carer would be inappropriate where, for example, the dementia is very severe or that an approach may increase distress and; 4) any other important reason that the key professional may have for why the person with dementia or their carer should not be contacted.

Every effort will be made to include those who meet the inclusion criteria but may not adequately understand verbal explanations or written information given in English. Where applicable, an independent and qualified translator/interpreter will be sought and invited to assist.

### Outcome measurements

#### Primary outcome

We derived our primary outcome measure and the effect size from discussions with practitioners in the feasibility phase of the trial. The consensus was that the clinical tasks involved in providing good quality care required at least two encounters per year, and that the educational intervention would promote this effectively in a majority of those in the intervention arm. Our hypothesis is, therefore, that in the intervention arm, the proportion of patients receiving two dementia-specific management reviews per year will increase between groups of patients by 50%, i.e. 20% (control) versus 70% (intervention), after the introduction of the educational intervention. Data relating to dementia-specific patient reviews and consultations relevant to dementia management will be extracted from the practice records.

#### Secondary outcome

1) Concordance with guidelines.

We will transcribe and scrutinise manual and electronic records for the recording of actions considered to be best practice in the diagnosis and management of dementia in primary care by using the NICE/SCIE dementia guidelines [2]. Appendix 1 shows the components of good practice that will be captured. The index consultation is the one where the suspicion of possible dementia is first recorded (Appendix 2).

2) Measurement of unmet needs in patients and carers.

Unmet needs will be captured using a questionnaire developed and validated by us in a previous trial [18].

### Sample size

Based on the study having 90% power to detect as significant, using a 5% 2-tailed significance level, a difference in the proportion of individuals with 2 or more dementia related GP visits of 50% (control: 20% versus Intervention: 70%) the required sample size, based on individual randomisation would be 23 per group - a total of 46 individuals. However, due to the use of cluster randomisation, the total required sample size needs to be inflated in order to take account of this clustering. The number of patients recruited per practice will also need to be inflated in order to maintain the sample sizes in the presence of attrition [19,20]. With 20 practices (10 per arm), the power to detect the differences postulated would be maintained if the ICC were of the order 0.37 or less. Thus the effective sample size with 10 patients per cluster and 20 practices would need to be 200. If the expected attrition rate were 1/3 (33.3%) then 15 patients would need to be recruited per practice in order to maintain the sample size of 10 patients per practice.

### Procedure

All practices will be asked to identify patients with symptoms of dementia by using electronic searches of their clinical record system updated by manual checks of the resulting list by medical and nursing staff. Where there is no informal carer eligible for interview, advice regarding consent for examination of medical records will be sought from an appropriate consultee. No personalised information will be retained following the record examinations, which will be carried out in the surgery by research team members who have honorary contracts with the relevant PCT.

This trial will last 36 months allowing for a follow-up period of 12 months that will capture effects on clinical practice, carer satisfaction, met and unmet needs. Data will be gathered before and 12 months after the introduction of the educational interventions.

#### Intervention

Participating practices will be randomised by an independent person to intervention or control arms using a computer generated randomisation programme. Practices randomly allocated to the intervention arm will be asked to participate in tailored learning activities consisting of an educational needs assessment followed by up to three face-to-face educational workshop sessions on dementia over a three-month period and will be given electronic resources which they can use during and after consultations with people with known or suspected dementia syndrome. Each educational workshop will be arranged at dates/times convenient to the practice team.

An experienced general practitioner with a background in postgraduateeducation will facilitate the small group workshops with the practice team. Tailoring of the education programme is carried out in a three step process: 1) in the first workshop, an educational needs analysis is undertaken using a standard checklist to identify aspects of dementia care which the practice perceives as problematic for them; 2) a prescription to meet the practice team's educational needs is then written to address shortcomings and sorting out the appropriate written educational resources, and; 3) the best forms of learning are then identified with a delivery and discussion with the team of the selected educational resource materials in the light of their perceived needs.

The 'normal care' arm will be given a summary of the NICE/SCIE dementia guidelines [2] and offered workshop training and electronic tools and resources at the end of the study. General practitioners, practice nurses and any other staff involved will be invited to give feedback on the educational resources.

Practices will be offered financial reimbursement to cover the use of locums and data collection costs (e.g. recruitment of patients and carers). Payments will be based on a sliding scale according to the number of partners in the practice, up to a maximum allocation.

#### Consent

People with dementia will be identified by practices and their lead clinician will check whether they fulfil the inclusion criteria. Where the lead clinician believes that the individual should not be approached (e.g. because they are receiving palliative care) they will be excluded. Before seeking consent from patients to participate in the study, practitioners will be asked for their opinion about the capacity of the person with dementia to give informed consent, using the Mental Capacity Act [21] as the framework for their judgement.

For patients judged as having capacity, the following information will be posted to the person with dementia: 1) a covering letter explaining the involvement of the practice and signed by the lead clinician; 2) a participant information sheet and; 3) a response letter and pre paid envelope to be returned to the research team. A researcher will arrange to see those patients with dementia and their carers who express an interest in participating in the study. This encounter can take place at the patient's home or in the practice, as they wish. Its purpose is to seek consent from the carer and consent/assent from the person with dementia for their participation in the study and allowing access to patient records.

For those judged by the lead clinician as lacking capacity to give informed consent, a consultee as outlined in the Mental Capacity Act [21] will be identified and consulted about possible involvement of the person with dementia in the trial. The relevant clinician will write to the consultee providing full information about the trial and asking whether or not they consent on behalf of the patient to enrolment.

Every effort will be made to identify a consultee for those judged to lack capacity to give informed consent. In the event that a consultee cannot be identified, the person with dementia will be excluded from participation in the trial. Figure 2 shows the process of identifying participants and how these will be approached, recruited and consented/consulted about EVIDEM-ED.

The process for obtaining participant informed consent or assent and guardian informed consent will be in accordance with the REC guidance [22], the Mental Capacity Act [21] and Good Clinical Practice (GCP) [23]. The investigator or their nominee and the participant or other legally authorised representative shall both sign and date the Informed Consent Form before the person can participate in the study. The participant will receive a copy of the signed and dated forms and the original will be retained in the Trial Master File. A third copy will be filed in the participant's medical notes and a signed and dated note made in the notes that informed consent was obtained for the trial.

With the informed consent of the person with dementia or their consultee, members of the research team will examine medical records in the surgery, using the themes shown in Appendix 1 to guide data extraction. No personalised information will be recorded or retained by the researcher and each case will be allocated a unique study number for the purposes of recording data. All other information will be stored in accordance with the Data Protection Act.

### Statistical analyses

We will assess the effect of the interventions at the practice level because the data will be cluster based and analyses will be performed on an intention to treat basis. Analyses of all quantitative responses will be performed with a general linear model with the arm and time as fixed effects and practice identity as a random effect.

We will analyse differences in detection rates by using binary logistic regression to include the cluster effect. These will be calculated before and after the intervention, excluding cases previously diagnosed in another practice. Concordance scores for diagnosis and management before and after the intervention will not directly be comparable as they comprise counts of actions taken over two different lengths of time. We will use Vickers' method and examine differences in baseline concordance scores across the arms of the study and then repeat the analysis for scores after the intervention. This analysis will also incorporate the cluster effect.

If outcome data are missing, we will assume they are "missing at random" (MAR). This means that the probability of missing data can be predicted by variables measured at baseline. In this case, an analysis which adjusts for the baseline predictors of 'missingness' (at least baseline response and treatment) will give an unbiased estimated of the treatment effect, making multiple imputation unnecessary. Multiple imputation will be used only if important baseline predictors are missing. Methods will then be employed which take account of the clustered nature of the data

### Trial management

The trial will be led by the Principal Investigator, Prof. Steve Iliffe and managed by the Programme Manager Ms. Jane Wilcock. The trial co-ordinating centre is the Department of Primary Care and Population Health at University College London. A Trial Management Committee will meet every two months to review the progress of the trial and its members will be recruited from; 1) the research teams in the EVIDEM programme and; 2) patient and public representatives working with the EVIDEM programme. A Trial Steering Committee (TSC) with a majority of members independent of the EVIDEM programme will meet six monthly to review the study. Because there were no adverse outcomes in the earlier trial of educational interventions, we propose not to have a data management committee but to give the chair of the TSC powers to convene a data management sub-group should there be any need to consider adverse events.

### Stopping rules and discontinuation

Participants can chose to leave the trial at any point. The trial will be terminated if it is shown to have a negative impact on routine medical care.

### Duration of the trial

The Trial will run for 36 months depending on the speed of recruitment of practices. Practices randomly allocated to receive training will receive this within three months period, with data collection at baseline and again at 12 months following baseline assessment.

### Participant involvement

Practices will receive regular updates on the trial's progress via newsletters and other media and will also be invited to annual summer schools and seminars run by the EVIDEM programme. This will include receiving study findings at the end of the Trial. Practices allocated to the control arm will receive training after the data collection period. The people with dementia and carers who participate will be offered an option to receive a lay version of the study outcomes and be invited to a lay conference at the end of the study period.

### User and public involvement

All user and carer representatives from the NICE/SCIE guideline development group have agreed to join the Reference Group that meets annually to provide critical evaluation of the EVIDEM programme by which the EVIDEM-ED study is overseen. They will bring their experience of the guideline development process and in particular their awareness of the specific gaps in evidence and the methodological problems of dementia research in the community. Members of the Reference group also include representatives from the Alzheimer's Society, the Council for Palliative Care, Age Concern England, the Association of London Government, Dementias and Neurodegenerative Diseases Research Network (DeNDRoN), national co-ordinating centre and the Mental Health Trust.

### Adverse events

We do not anticipate any unfavourable and unintended signs, symptoms, syndromes or illness to be caused to patients by general practitioners' participation in the trial. However, it is possible that an unknown serious condition, treatment or behaviour could come to the attention of the researchers.

Participants will be asked to contact the study site immediately in the event of any serious adverse event. All adverse events will be recorded and closely monitored until resolution or stabilisation, or until it has been shown that the study involvement is not the cause. The Chief Investigator shall be informed immediately of any serious adverse events and shall determine seriousness and causality in conjunction with any unexpected outcome of trial participation, together with the chair of the TSC.

In the event of a hitherto unknown severe or serious condition or situation becoming apparent to the research team, this will be discussed with the Chief Investigator who will notify the appropriate service as required. The Chief Investigator shall be responsible for all adverse event reporting. Any participant who experiences an adverse event may be withdrawn from the study at the discretion of the Investigator, but in consultation with the patient, their carer (where appropriate) and their general practitioner.

### Ethical issues

This study has particular ethical implications. Gaining consent from people with dementia raises complex issues and full research governance processes will be followed to gain meaningful informed consent, or where necessary, consult with those who are able to act as decision-makers. We have obtained ethical permissions from the NHS ethical review system and relevant NHS governance departments. We shall follow local adult protection procedures in each of the localities of the research and data collection.

### Ethics committee and regulatory approvals

The trial will not be initiated before the protocol, informed consent forms and participant and GP information sheets have received approval/favourable opinion from the Research Ethics Committee (REC) and the respective National Health Service (NHS) Research and Development (R&D) department. Should a protocol amendment be made that requires REC approval, the changes in the protocol will not be instituted until the amendment and revised informed consent forms and participant and GP information sheets (if appropriate) have been reviewed and received approval/favourable opinion from the REC and R&D departments. A protocol amendment intended to eliminate an apparent immediate hazard to participants may be implemented immediately providing that the REC are notified as soon as possible and an approval is requested. Minor protocol amendments only for logistical or administrative changes may be implemented immediately and the REC will be informed.

## Discussion

The EVIDEM-ED trial progresses from an earlier study and is developing and testing an intervention that is customised to the educational needs of individual practices. The deliverables from this programme will include an educational intervention for general practice and practice nursing combining timely dementia diagnosis and psychosocial support around the period of diagnosis with components appropriate to later stages of the disease trajectory. It will also include electronic resources on the same themes together with shared care guidelines for medication use. All deliverables will be made freely available to the NHS within the timeframe of the study.

The limitations that we anticipate are that the study is taking place in the South East of England, that participant practices will be more likely to be innovative early adopters than typical of general practice, and that local educational programmes developed to implement the National Dementia Strategy may influence practice activities. However, the results of this study may have wider implications, particularly about the value of tailoring educational interventions. The findings may be widely applicable to general practice and, if the trial shows benefit, could be utilised nationally within primary care as part of the implementation of the National Dementia Strategy [3].

## Competing interests

The authors declare that they have no competing interests.

## Authors' contributions

SI and JW developed the research proposal and obtained funding for the project. MG and PJ refined the protocol. IT-B and SI expanded and reworked the protocol to fit TRIALS's requirements, FL and TK field-tested the intervention and commented on the protocol. All authors read and commented on subsequent drafts of this paper and approved the final draft.

## Appendix 1

Semi-structured checklist to guide discussion in the educational needs assessment.

### Questions

1. How would you rate your current care for people with dementia and their carers (using a simple scale of good enough/satisfactory/needs substantial improvement)?

2. What grounds or criteria is your rating based on?

3. Does the number of people in your practice diagnosed with dementia correlate with the local prevalence figures?

4. How do you arrive at your decision for diagnosis of dementia?

5. How many older people with suspected dementia did you refer last year?

6. After diagnosis, what follow-up do you provide to people with dementia and their carers?

7. Are you using a shared care protocol for cholinesterase inhibitors? If 'yes', then: (i) who was involved in producing the protocol; (ii) who is involved in it implementation (e.g. hospital consultants, CPNs, Care of Older People team)

8. How effective do you think cholinesterase inhibitors are and how effective have you found them in your practice?

9. What non-pharmacological alternatives do you have available to help your patients (and their carers)

10. Based on your experience, what do you think are the important quality markers in caring for people with dementia? (What would you want for yourself?)

11. Is there anything that you would like improve about your current practice with patients with dementia? If yes, what is it and why would you like it to change?

## Appendix 2

Secondary outcome indicators; components of good practice.

### Diagnosis concordance

1. Request for blood tests at or after index consultation but before formal diagnosis

2. History of patient's symptoms documented at or after index consultation but before formal diagnosis

3. Cognitive testing at or after index consultation but before formal diagnosis

4. Depression screen used at or after index consultation but before formal diagnosis

5. Possible diagnosis discussed with patient or carer, or both

6. Referral to specialist service (including memory assessment unit) at or after index consultation

### Management concordance

1. Concerns of carer explored and documented

2. Behavioural and psychological symptoms elicited

3. Depression assessment or treatment documented

4. Referral to, or involvement of, social services

5. Referral to, or involvement of, voluntary organisations

6. Cholinesterase inhibitor drugs considered

7. Review of medication documented.
